# Long non-coding RNA CCDC144NL-AS1 promotes cell proliferation by regulating the miR-363-3p/GALNT7 axis in colorectal cancer

**DOI:** 10.7150/jca.65885

**Published:** 2022-01-01

**Authors:** Yue Zhang, Chaofan Peng, Jie Li, Dongsheng Zhang, Chuan Zhang, Kangpeng Jin, Dongjian Ji, Wen Peng, Junwei Tang, Yifei Feng, Yueming Sun

**Affiliations:** Department of General Surgery, The First Affiliated Hospital of Nanjing Medical University, Nanjing, Jiangsu, China.

**Keywords:** CCDC144NL-AS1, miR-363-3p, GALNT7, cell proliferation, colorectal cancer

## Abstract

Colorectal cancer (CRC) is a burdensome health concern worldwide. Long non-coding RNA (lncRNA) have emerged as vital roles in multiple cancers, including CRC. Increasing evidence has demonstrated that lncRNA CCDC144NL-AS1 acts crucial roles in tumor developments. Nevertheless, its role in CRC remains largely unknown. The level of CCDC144NL-AS1 expression was detected in 100 CRC tissues and paired adjacent tissues. The gain- and loss-of-function experiments were conducted to investigate the biological functions of CCDC144NL-AS1 *in vitro* and *in vivo*. The potential mechanism of CCDC144NL-AS1 exerting as competing endogenous RNAs (ceRNAs) was demonstrated by bioinformatics, luciferase reporter assay and *in vitro* experiments. CCDC144NL-AS1 was up-regulated in CRC tissues and cells. High CCDC144NL-AS1 was connected with the adverse clinicopathological features and worse prognosis of CRC. Furthermore, knockdown of CCDC144NL-AS inhibited the cell proliferation and led to the cell cycle G0-1/S arrest, whereas upregulated CCDC144NL-AS1 obtained the inverse results. Further study found that CCDC144NL-AS1 functioned as ceRNAs in regulating CRC proliferation. MiR-363-3p was the target of CCDC144NL-AS1, which sponges GALNT7 in regulating cell growth of CRC. The study demonstrated that the CCDC144NL-AS1/miR-363-3p/GALNT7 axis exerts on key roles in cell proliferation and presents an emerging target for CRC therapy and prognostic biomarker.

## Introduction

Colorectal cancer (CRC) is major burdensome health problems worldwide, with an evaluative 1,800,000 cases diagnosing and 0.8 million cases dying in 2018 1. In China, CRC also raises a critical health concern for its high incidence and mortality rate 2. In spite of the tremendous progress achieved in the treatment of CRC under great attention 3, 4, the complex molecular regulatory mechanisms of CRC are still unclear. Hence, it is crucial to further explore the unknown mechanisms of CRC progression.

Long non-coding RNAs (lncRNAs) are non-coding RNA consisting of >200 nucleotides with limited or no protein‐coding ability 5. It has been found lncRNA abundantly distributed in the nucleus and cytoplasm of mammalian and plant cells 6, 7. These lncRNAs can participate in multiple cellular processes, including cell growth, metastasis and differentiation 8, 9. Their regulation of genes is common at epigenetic, transcriptional and post transcriptional levels 10, 11. Increasing evidence showed that lncRNA can play the crucial role in a variety of tumors, including CRC 12-14. CCDC144NL-AS1 was a lncRNA discovered in recent years. Despite few reports about it, CCDC144NL-AS1 was investigated to act key roles in the multiple biological regulation, like positive regulation of endometriosis and inhibition of naïve-like state conservation of human pluripotent stem cells 15, 16. It has also been reported to act as an oncogene in gastric cancer 17, osteosarcoma 18 and ovarian cancer 19. In CRC, CCDC144NL-AS1 was predicted that could be involved in the copy number variations, which contributed to the poor prognosis of patients 20. However, the expression and function of CCDC144NL-AS1 in CRC are still unknown.

In this study, CCDC144NL-AS1was first detected and analyzed in CRC. Then we further explored the biological roles and mechanisms of CCDC144NL-AS1 in cell proliferation of CRC. All data suggested that CCDC144NL-AS1 is a tumor inhibitor of CRC and may be promising biomarker for diagnosis and molecular therapeutic targets in CRC.

## Material and methods

### Clinical specimen

Total 100 CRC tissues and adjacent tissues were obtained from the First Affiliated Hospital of Nanjing Medical University (NMU). All cases were between 18 and 75 years old, and were confirmed as primary colorectal adenocarcinoma by postoperative pathology. Patients who had received radiotherapy or chemotherapy before operation were excluded. All samples were frozen at -80 °C for long-term preservation. All patients signed informed consent before specimen collection. This research was ratified by the Ethics Committee of NMU.

### Cell lines and culture

Five CRC cell lines (HCT116, LoVo, SW480, DLD-1 and HT29) and normal intestinal epithelial cell (NCM460) were obtained from the Cell Bank of Type Culture Collection of the Chinese Academy of Sciences (Shanghai, China). SW480 and DLD-1 were cultured in RPMI 1640 (Winsent, Canada); HCT116, LoVo, HT29 and NCM460 were maintained in Dulbecco's modifed Eagle's medium (Winsent, Canada). All the medium replenished with 10% fetal bovine serum in a 5% CO2 humidified incubator at 37 °C.

### qRT-PCR and RT-PCR of microRNA

The procedures were referred to the publication 21. The specific primer for qRT-PCR and RT-PCR used in the study were shown in supplementary Table 1.

### Small interfering RNA transfection and lentiviral vectors

Inhibition and overexpression of CCDC144NL-AS1 was conducted by using a plasmid synthesized in GeneChem Corporation (Shanghai, China), and the empty vector was used as the negative control. Transfections of siRNA were carried out using Lipofectamine3000 (Invitrogen, USA) following the manufacturer's instructions, and their transfection efficiency were analyzed after 48 hours by qRT-PCR. A lentivirus expressing a small hairpin RNA (shRNA) against CCDC144NL-AS1 and negative control shRNA were designed by GeneChem Corporation (Shanghai, China). Puromycin was used to select for stable clones after 48 hours.

### Western blotting

Procedures were as described previously 21. The information about all antibodies used in the study is as follows: GALNT7 (Abcam, ab254971), GAPDH (Abcam, ab181602), cyclin-dependent kinase 4 (CDK4) (Abcam, ab108357), Cyclin D1 (Abcam, ab16663), p27 (Abcam, ab32034).

### Cell viability assay

The Cell Counting Kit-8 (CCK-8) (Beyotime, Shanghai, China) reagent was added into the cells for 120 min, then detect cell viability. 450 nm wavelength was chosen to detect.

500 differentially treated cells were added in a 6-well plate. The detailed procedures were adopted from the publication 22.

### 5-Ethynyl-2′-deoxyuridine (Edu)

Detailed procedures were described in previous publication 22. Nikon microscope (Nikon, Japan) captured the images.

### RNA fluorescent *in situ* hybridization (FISH)

The paired FISH kit (RiboBio, China) and the probe of CCDC144NL-AS1 were designed and synthesized by RiboBio (Guangzhou, China). Images were snapped by a confocal microscopy.

### Cell cycle

The treated cells were supplemented with 75% ethanol at 4 °C at least 12 h. Before detection, cells were washed twice using PSB after the separated ethyl alcohol removed, and then were stained with corresponding cell cycle detection reagents for 30 min in avoid of light. Finally, the cell cycle distribution was analyzed by FACSCalibur flow cytometer with CellQuest software (BD Biosciences).

### Dual-Luciferase reporter assay

The procedures were performed as before described 21. The wild-type and mutant CCDC144NL-AS1/GALNT7 sequences and 3ʹ-UTR of miR-363-3p were synthesized by GeneScript (Nanjing, China).

### Animal experiments

The animal study was ratified by the Animal Ethics Committee of NMU. For the tumorigenicity studies, different pretreated LoVo and DLD-1 cells were subcutaneously injected into the 4-week old BALB/c nude mice. Every five days, tumor volume was measured. A month later, the tumor tissues were surgically removed after mice euthanasia, then the tumor weight and the expression level of Ki-67 were detected.

### Immunohistochemistry (IHC)

The experimental process of IHC was previously described 23. The paraffin‑embedded tissue sections were deparaffinized in xylene, rehydrated in alcohol and distilled water. Then, the sections were washed in phosphate-buffered saline, placed in H_2_O_2_ in the dark, soaked in 5% bovine serum albumin. Finally, the tissue sections were reacted with anti-Ki67 antibodies (Abcam, USA) overnight, followed by incubation with secondary antibody conjugated with HRP. The sections were observed with an inverted microscope (Nikon, Japan). The Ki-67 staining intensity was graded as 0 (no staining), 1 (weak), 2 (moderate), and 3 (strong). The proportion of Ki-67 positive cells was scored as 0 (<10%), 1 (10-50%), 2 (>50%). Score was calculated as positive rate score multiplied intensity score.

### Statistical analysis

SPSS 22.0 and GraphPad Prism 8.0 were used to analyze the data. Each experiment was conducted in tripe. Student's t-test, Chi-square test and Kaplan‑Meier analysis were used to conduct the data analysis and survival comparison.* P*<0.05 was significant.

## Results

### CCDC144NL-AS1 elevated in CRC and correlated with adverse clinical features

**Figure 1:**
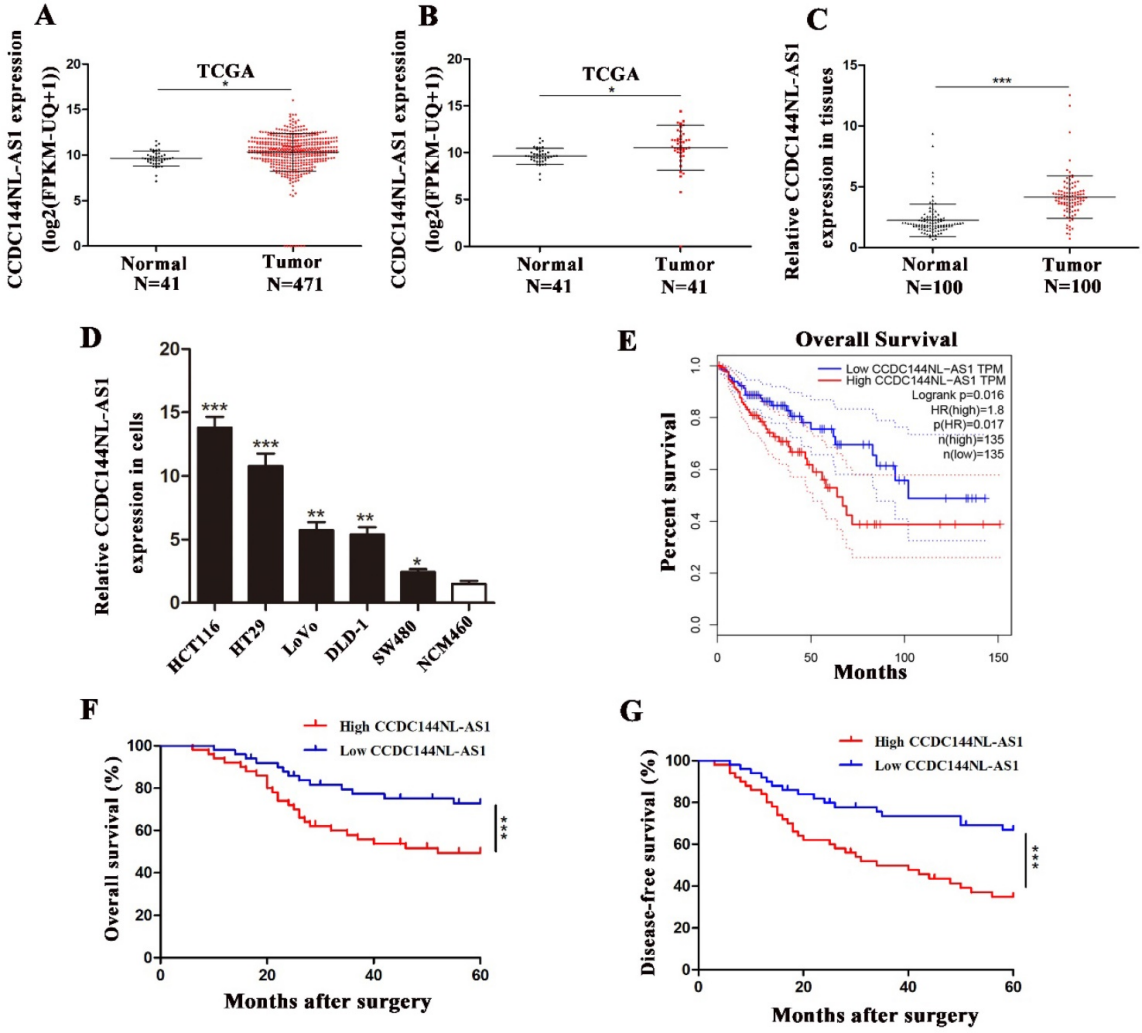
** CCDC144NL-AS1 was upregulated in CRC and predicted poor prognosis. A and B.** Relative expression of CCDC144NL-AS1 in unpaired and paired COAD from the TCGA database. **C.** Relative expression of CCDC144NL-AS1 in 100 CRC tissues and matched adjacent normal tissues. **D.** Relative expression of CCDC144NL-AS1 in CRC cells and normal colon epithelial mucosa cell line (NCM460). **E.** Online Kaplan-Meier overall survival (OS) curves based on the CCDC144NL-AS1 expression in COAD. **F and G.** OS and disease-free survival (DFS) of 100 CRC patients based on CCDC144NL-AS1 expression by Kaplan-Meier survival analysis. *p < 0.05, **p < 0.01, ***p < 0.001.

We first analyzed CCDC144NL-AS1 in TCGA database, which contained data of 471 colon adenocarcinoma (COAD) samples and 41 normal tissue samples. The result suggested that CCDC144NL-AS1 increased both in unpaired tissues (Fig. 1A) and paired tissues (Fig. 1B) of COAD in TCGA database. Then, 100 CRC tissues and adjacent tissues was used to confirm the level of CCDC144NL-AS1 expression by qRT-PCR, and results showed that CCDC144NL-AS1 was increased in CRC tissues in comparison with adjacent tissues (Fig. 1C). Further detection presented that CCDC144NL-AS1 was also elevated in CRC cell lines in compared with colon epithelial mucosa cell line NCM460 (Fig. 1D). Due to CCDC144NL-AS1 in LoVo and DLD-1 was in the middle expression of all CRC cell lines, they were chosen for subsequent study. By further analyzing the relationship between CCDC144NL-AS1 expression and clinicopathological information, we discovered that higher CCDC144NL-AS1 predicted worse clinical pathological features (Table 1). Moreover, the online Kaplan Meier plot (GEPIA) indicated that a higher CCDC144NL-AS1 expression level predicted poorer overall survival in COAD (Fig. 1E). Based on follow-up information of the 100 patients, we found that upregulation of CCDC144NLAS1 was connected with worse overall survival (OS) and disease-free survival (DFS) in CRC by using Kaplan-Meier curves (Fig. 1F and G). Abovementioned results showed that CCDC144NL-AS1 possibly played an oncogenic role in CRC.

### Knockdown of CCDC144NL-AS1 inhibits cell proliferation of CRC *in vitro*

**Figure 2:**
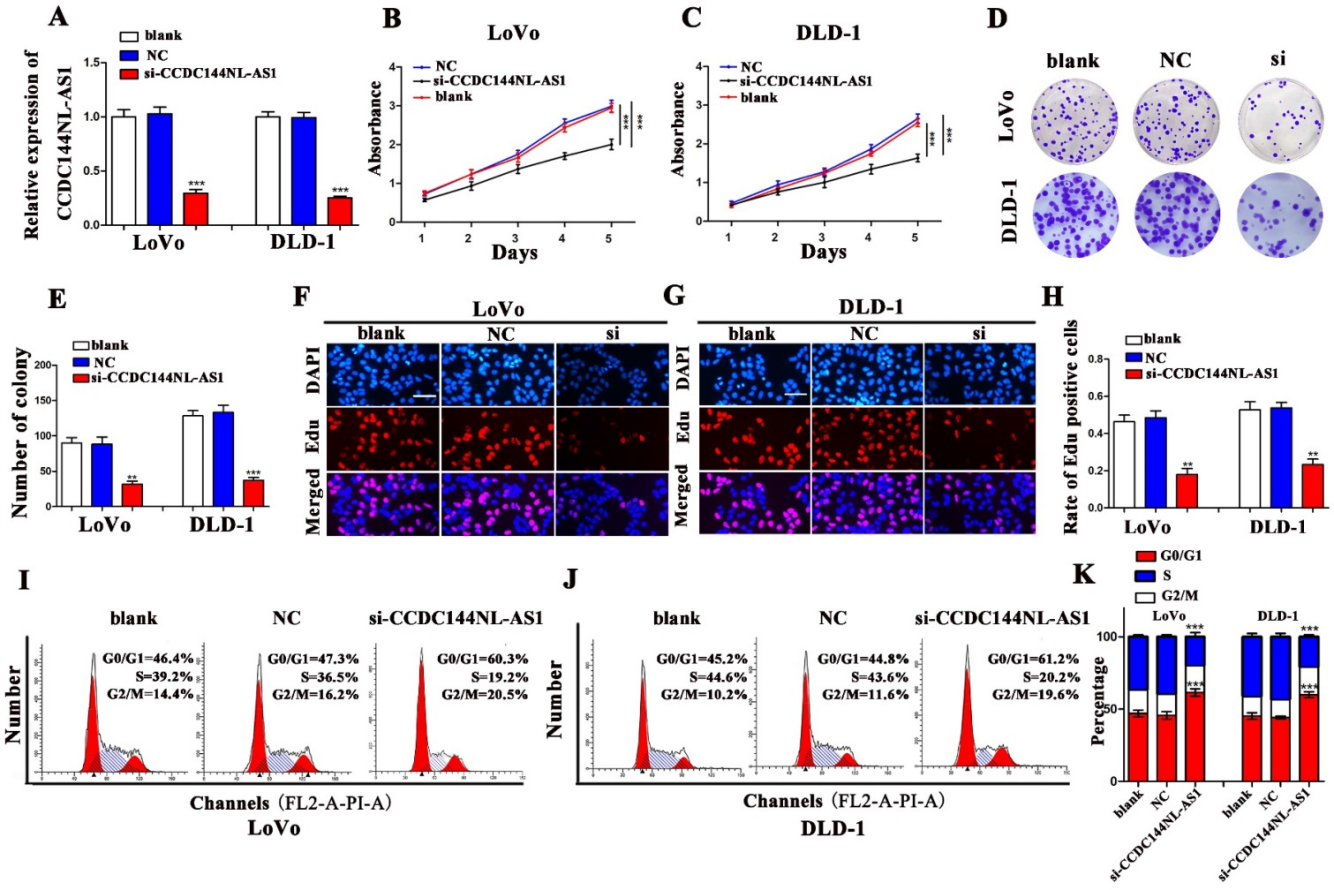
** Knockdown of CCDC144NL-AS1 inhibited CRC cell proliferation and caused cell cycle G0-1/S arrest. A.** Relative CCDC144NL-AS1 expression in LoVo and DLD-1 cells treated with si-CCDC144NL-AS1 transfection. **B and C.** Effects of CCDC144NL-AS1 silencing on the cell viability were detected by CCK‑8 assay in LoVo and DLD-1 cells. **D-H.** Effects of CCDC144NL-AS1 silencing on cell proliferation were measured by the colony formation assay and the Edu assay in LoVo and DLD-1 cells. Scale bar: 50 µm. **I-K.** Effects of CCDC144NL-AS1 silencing on cell cycle distribution in LoVo and DLD-1 cells. **p < 0.01, ***p < 0.001.

CCDC44NL-AS1 were knocked down in LoVo and DLD-1 cells with a small interfering RNA (si-CCDC44NL-AS1), respectively. The qRT-PCR results presented that CCDC144NL-AS1 were significantly inhibited by si-CCDC144NL-AS1 both in LoVo and DLD-1 cells (Fig. 2A). Then the CCK-8 presented that downregulated CCDC144NL-AS1 markedly decreased the cell viability (Fig. 2B and 2C). As showed in Fig. 2D and 2E, silencing CCDC144NL-AS1 repressed the cell formative abilities of LoVo and DLD-1. The Edu assays confirmed the similar trends that knockdown CCDC144NL-AS1 dramatically inhibited the DNA synthesis process of LoVo and DLD-1 cells (Fig. 2F-H). Furthermore, the results of cell cycle distribution revealed that knockdown of CCDC44NL-AS1 could result in cell cycle arrest in G0-1/S phage (Fig. 2I-K).

### Overexpression of CCDC144NL-AS1 promotes cell proliferation of CRC

**Figure 3:**
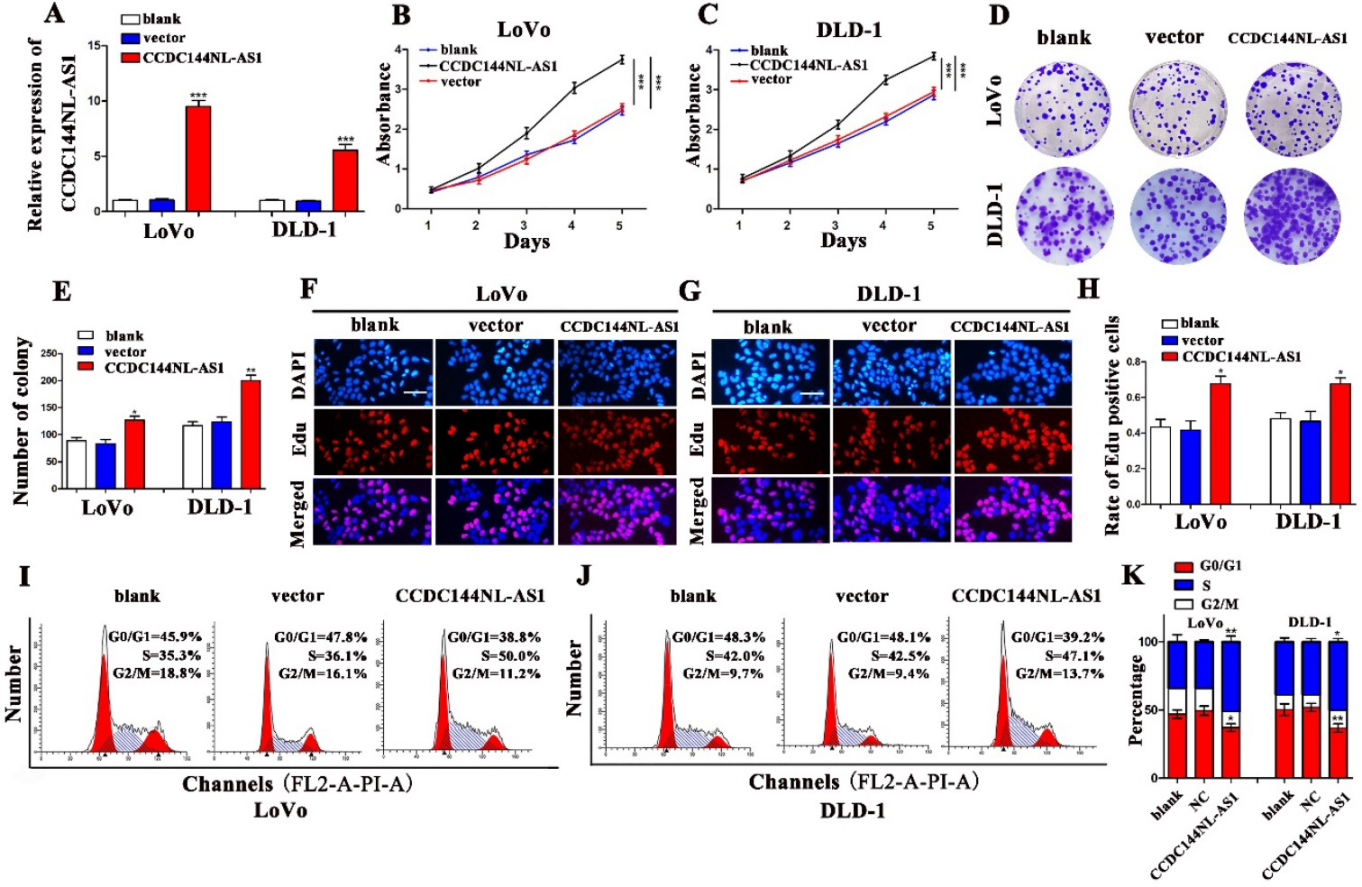
** Overexpression of CCDC144NL-AS1 promoted CRC cell proliferation and accelerated cell cycle G0-1/S transition. A.** Relative CCDC144NL-AS1 expression in LoVo and DLD-1 cells treated with CCDC144NL-AS1 overexpressing plasmid transfection. **B and C.** Effects of CCDC144NL-AS1 overexpression on cell viability were detected by CCK‑8 assay in LoVo and DLD-1 cells. **D-H.** Effects of CCDC144NL-AS1 overexpression on cell proliferation were measured by the colony formation assay and the Edu assay in LoVo and DLD-1 cells. Scale bar: 50 µm. **I-K.** Effects of CCDC144NL-AS1 overexpression on cell cycle distribution in LoVo and DLD-1 cells. *p < 0.05, **p < 0.01, ***p < 0.001.

To verify the function of CCDC144NL-AS1 in CRC cell proliferation, CCDC144NL-AS1 overexpressing plasmids were transfected to LoVo and DLD-1 cells, and result of qRT-PCR indicated that CCDC144NL-AS1 was dramatically elevated (Fig. 3A). Then, cell proliferation assays found that the results of CCDC144NL-AS1 overexpression and knockdown were completely opposite. The CCK-8 assays revealed that upregulation of CCDC144NL-AS1 markedly promoted cells viability compared with control vector (Fig. 3B and C). Then the assays of colony formation showed that overexpressed CCDC144NL-AS1 improved the cells formative abilities of LoVo and DLD-1 (Fig. 3D and E). The Edu assay suggested that elevated CCDC144NL-AS1 promoted DNA synthesis process in LoVo and DLD-1 cells (Fig. 3F-H). Cell cycle analysis showed that overexpression of CCDC144NL-AS1 accelerated cells from G0/G1 phase stepping to S phase (Fig. 3I-K). All results demonstrated that CCDC144NL-AS1 promoted LoVo and DLD-1 growth of CRC possibly by G0-1/S cell cycle regulation.

### Knockdown of CCDC144NL-AS1 represses tumor growth *in vivo*

**Figure 4:**
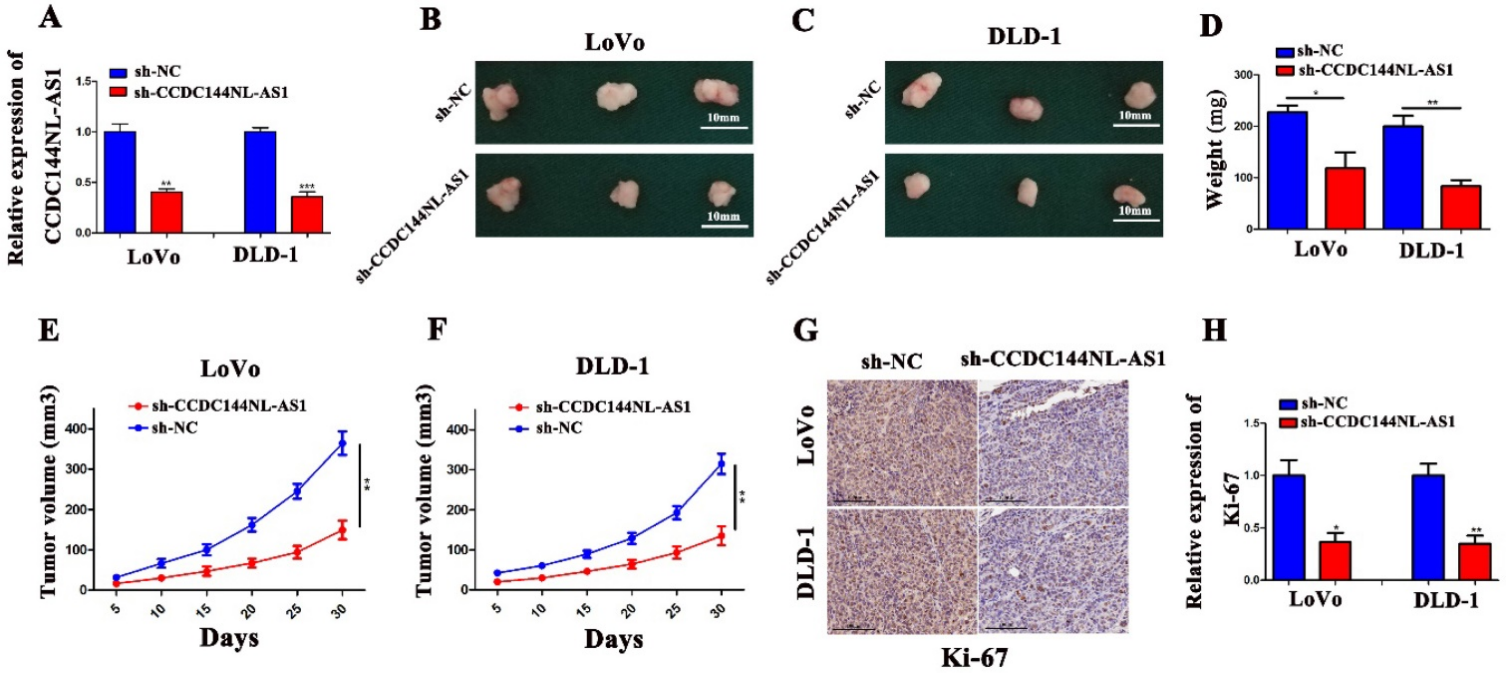
** Knockdown of CCDC144NL-AS1 inhibited CRC tumor growth *in vivo*. A.** Relative mRNA expression of CCDC144NL-AS1 in LoVo and DLD-1 cells following sh-CCDC144NL-AS1 transfection. **B.** Images of xenograft tumors in the nude mouse model under different treatments. **D-F.** Analysis of tumor weight and size in the sh-CCDC144NL-AS1 and control groups. **G and H.** The expression of Ki-67 from the xenografts were measured by IHC. *p < 0.05, **p < 0.01, ***p < 0.001.

We performed the tumor xenografting in nude mice to investigate the effects of CCDC144NL-AS1 in tumorigenesis *in vivo*. After transfection with CCDC144NL-AS1 inhibitor lentivirus (sh-CCDC144NL-AS1), the mRNA expression of CCDC144NL-AS1 were significantly downregulated (Fig. 4A). We found that the tumor weight and tumor size were markedly decreased with knockdown of CCDC144NL-AS1 both in LoVo and DLD-1 cells (Fig. 4B-F). Furthermore, we detected the expression of Ki-67 in mouse tissues by IHC. The result showed that tumors transfected with sh-CCDC144NLAS1 showed lower expression of Ki-67 in comparison with sh-NC groups (Fig. 4G and 4H). These results further indicated CCDC144NL-AS1 acts vital carcinogenic roles in the cell proliferation of CRC *in vivo*.

### CCDC144NL-AS1 exerts on a molecular sponge for miR-363-3p of CRC

**Figure 5:**
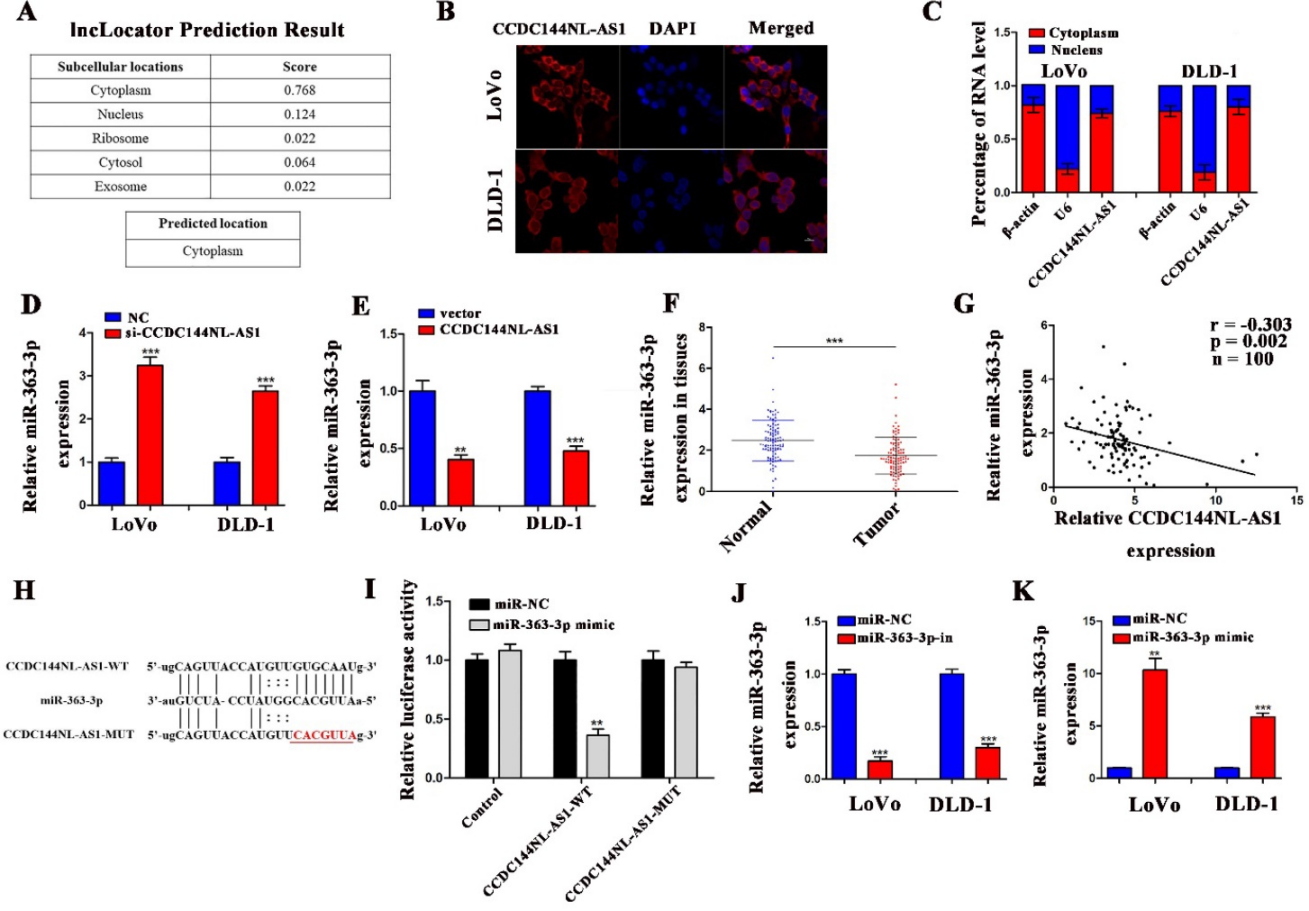
** CCDC144NL-AS1 acted as a molecular sponge for miR-363-3p in CRC cells. A.** Prediction of CCDC144NL-AS1 localization in cells by bioinformatics tools. **B and C.** Subcellular localization of CCDC144NL-AS1 in CRC cells detected by FISH and qRT-PCR. **D and E.** Effects of CCDC144NL-AS1 alteration on the expression of miR-363-3p detected by qRT-PCR in LoVo and DLD-1 cells. **F.** Relative expression of miR-363-3p in 100 CRC tissues and matched adjacent normal tissues. **G.** Correlation analysis of the expression of CCDC144NL-AS1 and miR-363-3p in 100 CRC tissues. **H.** The predicted binding sites between CCDC144NL-AS1 and miR-363-3p. **I.** Luciferase activity after MiR-363-3p mimics or miR-NC and pmirGLO-CCDC144NL-AS1-WT or pmirGLO-CCDC144NL-AS1-MUT co-transfected into LoVo cells. **J and K.** Relative expression of miR-363-3p in LoVo and DLD-1 cells treated with miR-363-3p inhibitor (miR-363-3p-in) and mimic. **p < 0.01, ***p < 0.001.

To investigate the potential mechanism by which CCDC144NL-AS1 influences the proliferation of CRC cells, we used CPAT tool to find that CCDC144NL-AS1 was mainly located in cytoplasm of human species (Fig. 5A). Then the FISH and qRT-PCR assays further validated that CCDC144NL-AS1 was located in CRC cytoplasm (Fig. 5B and 5C). Therefore, we speculated that CCDC144NL-AS1 possibly served as a ceRNA of miRNAs. Through bioinformatics analysis, we found that CCDC144NL-AS1 may be sponge of miR-363-3p. The results of qRT-PCR showed that miR-363-3p was upregulated after CCDC144NL-AS1 silencing (Fig. 5D), while was downregulated after CCDC144NL-AS1 overexpression (Fig. 5E). Then the mRNA expression of miR-363-3p was detected by qRT-PCR in the 100 paired of CRC tissues, and the results showed that it was significantly downregulated in tumor tissues (Fig. 5F). Further analysis revealed that a negative correlation existed between CCDC144NL-AS1 and miR-363-3p (Fig. 5G). After transfection of miR-363-3p inhibitor (miR-363-3p-in) and miR-363-3p mimic, the expression of miR-363-3p was significantly downregulated and upregulated, respectively (Fig. 5J and 5K). To examine whether CCDC144NL-AS1 directly targeted miR-363-3p, we conducted a luciferase assay in LoVo cells, and the result showed that miR-363-3p dramatically repressed the luciferase activity of the wild-type CCDC144NL-AS1 3'UTR (Fig. 5H and 5I). All data revealed that CCDC144NL-AS1 may sponge miR-363-3p in CRC cells.

### CCDC144NL-AS1 promotes cell proliferation of CRC cells by miR-363-3p regulation

**Figure 6:**
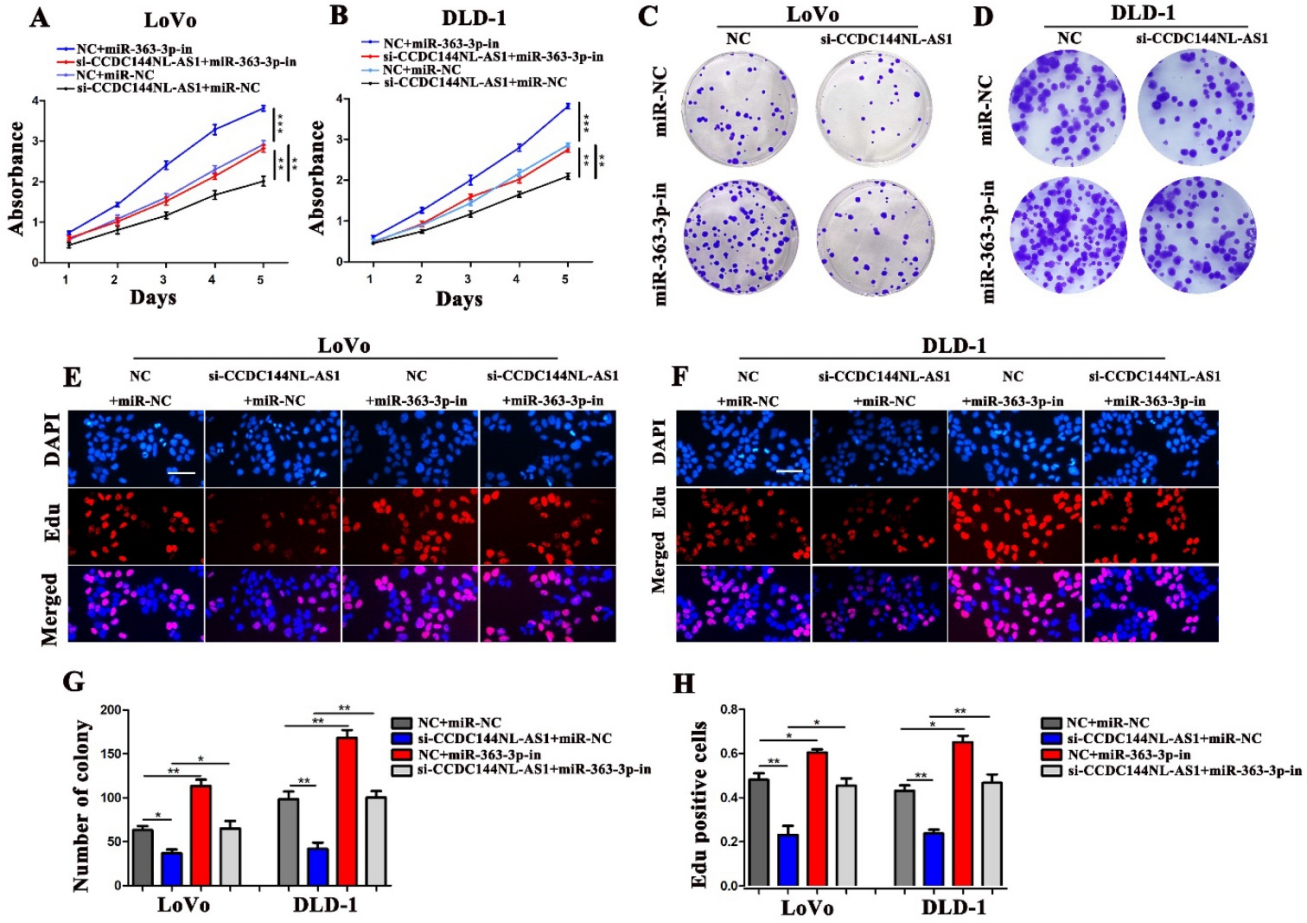
** The regulation of CCDC144NL-AS1 on CRC cell was mediated by miR-363-3p. A and B.** Cell viability of LoVo and DLD-1 cells in different groups was detected by the CCK‑8 assay. **C-H.** Cell proliferation of LoVo and DLD-1 cells in different groups was detected by the colony formation assay and Edu assay. Scale bar: 50 µm. *p < 0.05, **p < 0.01, ***p < 0.001.

To reveal whether the carcinogenesis of CCDC144NL-AS1 on CRC cells was mediated by miR-363-3p, LoVo and DLD-1 cells were co-transfected with si-CCDC144NL-AS1/NC and miR-363-3p inhibitor (miR-363-3p-in)/miRNA negative control (miR-NC). Then cell proliferation assays were performed, and the results showed that knockdown of CCDC144NL-AS1 alone significantly inhibited the cell proliferation as the previous outcome, and miR-363-3p silencing alone remarkably promoted the cell proliferation of CRC cells. Moreover, miR-363-3p silencing could dramatically attenuate the function of CCDC144NL-AS1 knockdown on cell proliferation (Fig. 6A-H). These data indicated that CCDC144NL-AS1 promotes cell proliferation on CRC by mediating miR-363-3p.

### GALNT7 was a target of miR-363-3p involved in CCDC144NL-AS1 induced cell proliferation

**Figure 7:**
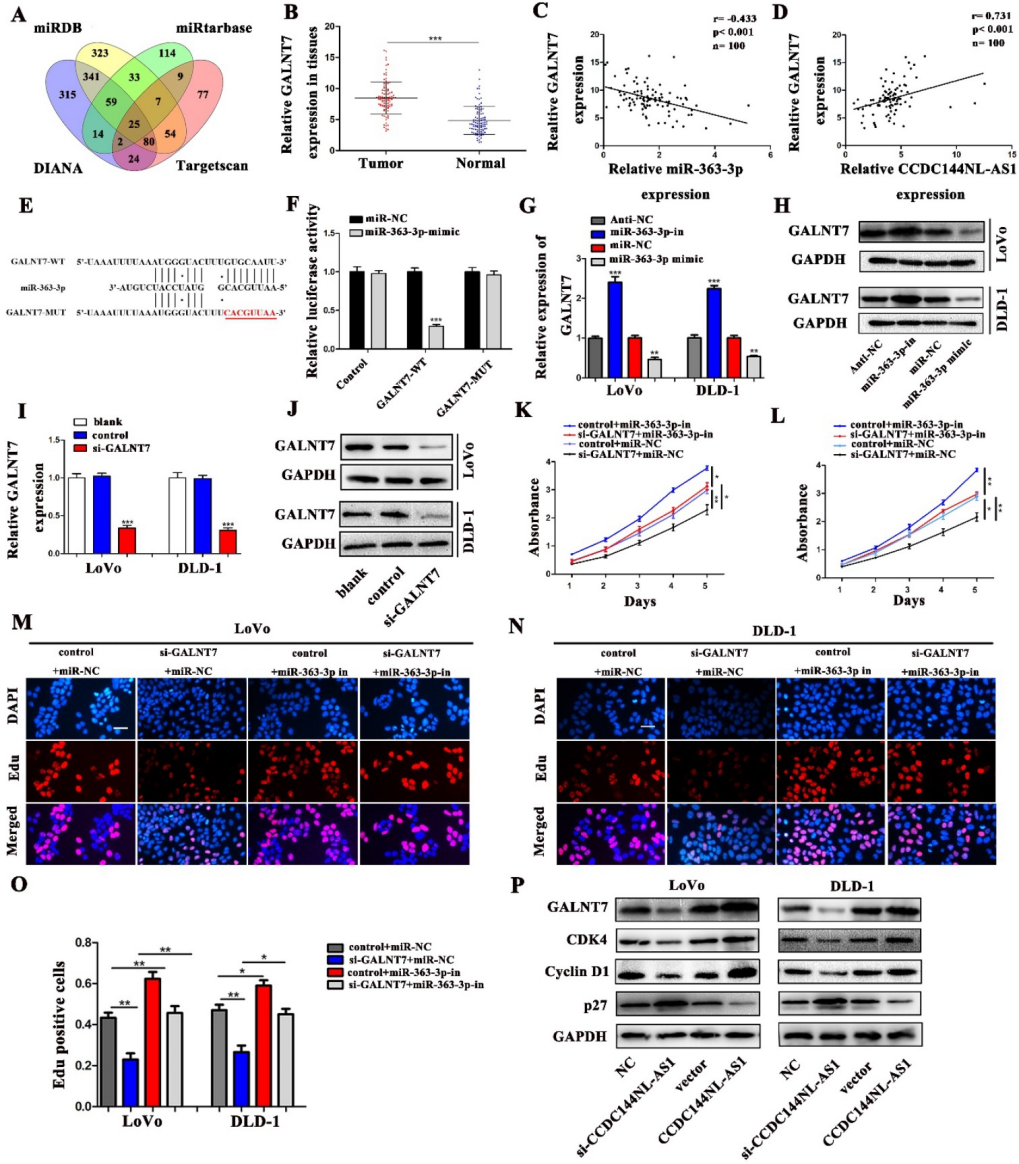
** GALNT7, a target gene of miR-363-3p, involved in CCDC144NL-AS1 regulation. A.** The potential target genes of miR-363-3p was predicted by miRDB, miRtarbase, DIANA and Targetscan database. **B.** Relative mRNA expression of GALNT7 in 100 CRC tissues and matched adjacent normal tissues. **C.** Correlation analysis of the expression of GALNT7 and miR-363-3p in 100 CRC tissues. **D.** Correlation analysis of the expression of GALNT7 and CCDC144NL-AS1 in 100 CRC tissues. **E.** The predicted binding sites between GALNT7 and miR-363-3p. **F.** Luciferase activity after MiR-363-3p mimics or miR-NC and pmirGLO-GALNT7-WT or pmirGLO-GALNT7 -MUT co-transfected into LoVo cells. **G and H.** GALNT7 expression was detected by qRT-PCR and western blotting after miR-363-3p inhibition and overexpression. **I and J.** GALNT7 expression was detected by qRT-PCR and western blotting after si-GALNT7 transfection. **K and M.** Cell viability of LoVo and DLD-1 cells in different groups was measured by the CCK-8 assay. **N and O.** Cell proliferation of LoVo and DLD-1 cells in different groups was measured by the Edu assay. Scale bar: 50 µm. **P.** The expression of GALNT7, CDK4, Cyclin D1 and p27 was detected in CRC cells by western blotting after CCDC144NL-AS1 knockdown and overexpression. *p < 0.05, **p < 0.01, ***p < 0.001.

**Figure 8:**
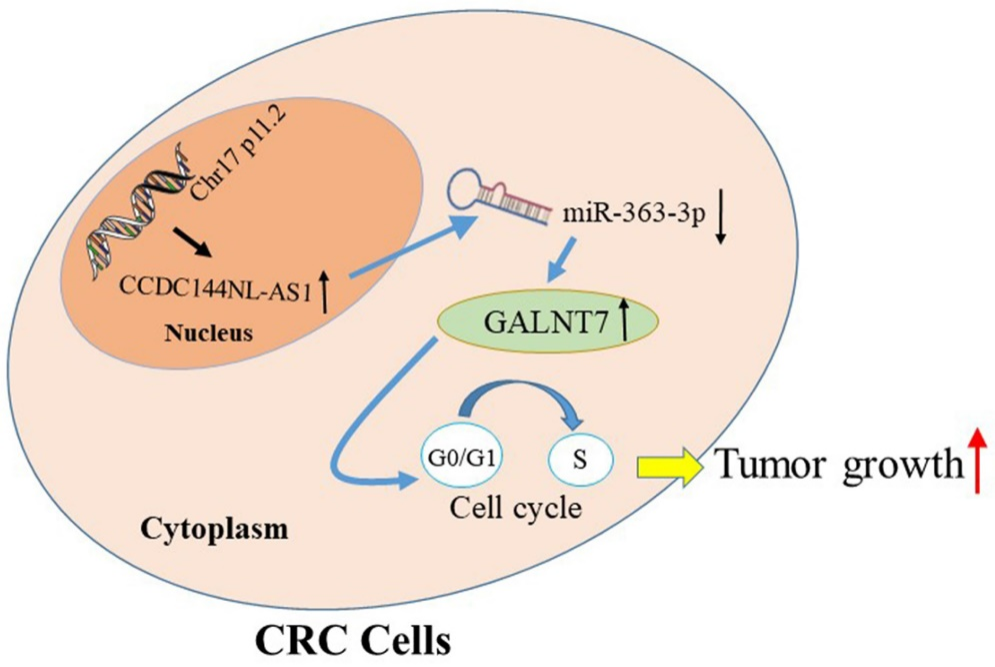
** The schematic model of CCDC144NL-AS1 in regulating CRC.** Increased CCDC144NL-AS1 competitively sponged more miR-363-3p, led to the negative effect of miR-363-3p on GALNT7 reduced, then accelerated the cell cycle G0-1/S transition and promoted cell proliferation in CRC cells.

To seek the targets of miR-363-3p in CRC, we used the bioinformatics data by miRDB, miRtarbase, DIANA and Targetscan databases. 25 candidate genes were discovered after the intersection of these databases, GALNT7 was contained (Fig. 7A). Through literature review, we found GALNT7 played an important role on the biological regulation of CRC cells, which was highly likely to be the target of miR-363-3p. To verify our speculation, GALNT7 was detected in these 100 paired CRC tissues and adjacent tissues, and the results presented that GALNT7 was overexpressed in CRC tissues in comparison with the adjacent tissues (Fig. 7B). Further analysis found that a remarkably negative relation between GALNT7 and miR-363-3p (Fig. 7C), and a positive correlation was between CCDC144NL-AS1 and GALNT7 in these 100 tumor tissues (Fig. 7D). Then analysis of luciferase assays confirmed that miR-363-3p remarkably restrained the luciferase activities in the wild type of GALNT7 3'UTR (Fig. 7E and 7F), indicating miR-363-3p could sponge GALNT7 in CRC cells. Then we detected the GLANT7 expression in CRC cells, the level of GALNT7 mRNA and protein was both downregulated with the upregulation of miR-363-3p, but increased with the downregulation of miR-363-3p (Fig. 7G and 7H). si-GALNT7 was transfected to LoVo and DLD-1 to investigate the relation between GALNT7 and miR-363-3p. GALNT7 was downregulated both on mRNA and protein levels after si-GALNT7 transfection (Fig. 7I and 7J). Then, LoVo and DLD-1 were co-transfected with miR-363-3p-in/miR-NC and si-GALNT7/NC. CCK-8 and Edu assays both revealed that downregulated GALNT7 not only repressed the cell growth, but also remarkably attenuated the function of miR-363-3p knockdown on cell inhibition (Fig. 7K-O). Furthermore, we detected GALNT7, CDK4, Cyclin D1 and p27 in CRC cells by western blot, we found GLANT, CDK4 and Cyclin D1 were downregulated and p27 was upregulated after CCDC144NL-AS1 knockdown. The similar results were also observed after CCD144NL-AS1 overexpression (Fig. 7P). All these results indicated that GALNT7 participated in the regulation of CCDC144NL-AS1 on the cell proliferation of CRC as the downstream of miR-363-3p, and the complete mechanism by which CCDC144NL-AS1 promoted CRC cell proliferation was shown in Figure 8.

## Discussion

Accumulating investigations revealed that lncRNAs play vital roles in human diseases, including various cancers 24-27. Through the analysis of TCGA database, we discovered that CCDC144NL-AS1 elevated in COAD, and higher CCDC144NL-AS1 expression predicted a poorer prognosis of patients. Our results first detected that CCDC144NL-AS1 was significantly increased in CRC. Upregulated CCDC144NL-AS1 was closely related to the worse clinicopathological features. Furthermore, elevated CCDC144NL-AS1 was connected with worse OS and PFS in CRC patients. Functional assays investigated that decreased CCDC144NL-AS1 repressed cell growth and arrested cell cycle, whereas overexpressed CCDC144NL-AS1 reversed the abovementioned results. Animal experiments further detected that decreased CCDC144NL-AS1 inhibited the cell proliferation. All data suggested that CCDC144NL-AS1 plays carcinogenic roles in CRC.

The ceRNAs are transcripts that can regulate each other at post-transcription by competing for shared miRNAs, which was named since 2011 28, 29. Sufficient evidence has showed that the functionalities of lncRNAs in tumorigenesis could be partially mediated by ceRNA crosstalk, including CRC 30, 31. For example, the oncogenic role of H19 was reported that attributed to its ceRNA activity to sponge miR-138 and miR-200a in CRC 32, 33. LncRNA UICLM was also founded that facilitated cell metastasis by serving as ceRNAs of CRC 34. Recently, the ceRNA role of CCDC144NL-AS1 has been discovered in gastric cancer 17. In combination with our result that CCDC144NL-AS1 mainly distributed in cytoplasm, we assumed that CCDC144NL-AS1 functions as ceRNAs in CRC. miR-363-3p may be the novel target of CCDC144NL-AS1 through serial assays. miR-363-3p mainly acted as a tumor inhibitor, including ovarian cancer 35, osteosarcoma 36, lung cancer 37, hepatocellular carcinoma 38 and CRC 39. It was involved in tumorigenesis, cell proliferation, invasion and metastasis by different regulatory mechanisms. Recently, its biological role of ceRNA was also determined in multiple cancers 35, 40. However, the relation between CCDC144NL-AS1 and miR-363-3p were unclear in CRC. MiR-363-3p was markedly decreased in CRC tissues, and negative correlations appeared between CCDC144NL-AS1 and miR-363-3p. *In vitro* results further verified that downregulated miR-363-3p dramatically attenuated the effects of downregulated CCDC144NL-AS1 on CRC cell inhibition. All results demonstrated that CCDC144NL-AS1 plays crucial roles by binding miR-363-3p in CRC.

The ceRNAs of lncRNAs mainly relies on the derepression of miRNAs' targets 41. The targets of miRNA are an indispensable part of ceRNA network. We discovered that GalNAc-transferase-7 (GALNT7) was the potential target of miR-363-3p through analysis and screening. Previous studies have demonstrated that aberrant expression of GALNT7 were involved in the developments of multiple tumors, such as glioma 42, cervical cancer 43 and CRC 44, by affecting cell proliferation, differentiation, invasion and migration. GALNT7 was also reported to be targeted by some miRNAs in various cancers, such as miR-123-5p and miR-154 43, 45. Moreover, GLANT7 has been reported to be involved in the ceRNA network as the target of miR-34a in CRC 44, 46. However, the relation between GALNT7 and miR-363-3p was never mentioned. In this study, we discovered GLANT7 was upregulated in CRC, which was accordance with previous literature. Further analysis presented that GALNT7 was positively connected to CCDC144NL-AS1, and negatively related to miR-363-3p. Moreover, *in vitro* results revealed that GLANT knockdown significantly attenuated the effects of downregulated miR-363-3p on CRC cell proliferation. Thus, these results suggested that GALNT7 may be the downstream of miR-363-3p, and the CCDC144NL-AS1/miR-363-3p/GALNT7 axis could promote cell proliferation in CRC. Furthermore, accelerated cell proliferation is usual linked to the aberrant regulation of cell cycle 47. During the whole process of cell cycle, G1/S transition was the crucial point that determines whether cells enter the process of proliferation 48. In the current study, we found that CCDC144NL-AS1 downregulation resulted in G0-1/S phase arrest, while CCDC144NL-AS1 overexpression led to G0-1/S phase acceleration. Further detection of hallmarks in G1/S phase, including CDK4, Cyclin D1 and p27, indicated that CCDC144NL-AS1 was a vital role in acceleration of G1/S transition in CRC cells. Hence, CCDC144NL-AS1 exerted as a ceRNA, may promote cell proliferation in CRC by accelerating G1/S transition. Even so, this study still has some limitations. In one respect, the deeper mechanism that how CCDC144NL-AS1/miR-363-3p/GALNT7 axis promoted cell proliferation in CRC waits to be excavated. In the other respect, the possible relationship between CCDC144NL-AS1 and tumor metastasis were never detected in this study, which need further investigate.

To conclude, we investigated that CCDC144NLA-AS1 was a novel oncogenic lncRNA in CRC. Then, we demonstrated that CCDC144NL-AS1 could facilitate cell growth and promote cells transition from G0-G1 phase to S phase though miR-363-3p-GALNT7 axis in CRC. The study suggested that CCDC144NL-AS1 may be potential targets of diagnosis and therapies for CRC.

## Supplementary Material

Supplementary table.

## Figures and Tables

**Table 1:** Expression of CCDC144NL-AS1 in CRC tissues and adjacent tissues

Clinical features	CCDC144NL-AS1 expression			
	n	high	low	P value
**Gender**		50	50	
man	62	32	30	0.680
female	38	18	20	
**Age (year)**				
>60	65	30	35	0.295
≤ 60	35	20	15	
**Tumor size**				
>5 cm	42	26	16	0.043*
≤5 cm	58	24	34	
**Depth of invasion**				
T1/T2	35	12	23	0.021*
T3/T4	65	38	27	
**Lymph node metastasis**				
Absent	39	14	25	0.024*
Present	61	36	25	
**Liver metastasis**				
Present	10	7	3	0.182
Absent	90	43	47	
**Location**				
rectal	58	28	30	0.685
colon	42	22	20	

*P<0.05.

## Abbreviations

CRC: colorectal cancer
lncRNA: long non-coding RNA
siRNA: small interfering RNA
shRNA: small hairpin RNA
CCK‑8: Cell Counting Kit‑8
Edu: 5‑ethynyl‑2'‑deoxyuridin
FISH: RNA fluorescent *in situ* hybridization
IHC: Immunohistochemistry
COAD: colon adenocarcinoma
ceRNA: competing endogenous RNA
GALNT7: GalNAc-transferase-7
