# Supplementary material for: Long non-coding RNA CCDC144NL-AS1 promotes cell proliferation by regulating the miR-363-3p/GALNT7 axis in colorectal cancer
Source: J Cancer. 2022 Jan 1;13(3):752–63. doi: 10.7150/jca.65885 (PMC8824904; doi:10.7150/jca.65885)
Supplement: Supplementary file 1 — Supplementary table. [file jcav13p0752s1.pdf]

Supplementary Table 1: The primers for qRT-PCR.

hsa-CCDC144NL-AS1	Forward Primer	AGCTGGAGATCACTTAGTGTAAGG
	Reverse Primer	AAGCTAGGCTTGTCTTTATTCCT
hsa-β-actin	Forward Primer	CATGTACGTTGCTATCCAGGC
	Reverse Primer	CTCCTTAATGTCACGCACGAT
hsa-GAPDH	Forward Primer	TGTGGGCATCAATGGATTTGG
	Reverse Primer	ACACCATGTATTCCGGGTCAAT
hsa-U6	Forward Primer	CTCGCTTCGGCAGCACA
	Reverse Primer	AACGCTTCACGAATTTGCGT
hsa-GALNT7	Forward Primer	GGTTCATCTTACGCAGTTTGCT
	Reverse Primer	GGGCATGGGGTCATTGACA
hsa-miR-363-3p	Forward Primer	CGGGCAATTGCACGGTATCC
	Reverse Primer	CAGCCACAAAAGAGCACAAT

The primers for RT-PCR

hsa-miR-363-3p	CCTGTTGTCTCCAGCCACAAAAGAGCACAATATTTCAGGAGACAACAGGTACAGAT
hsa-U6	AACGCTTCACGAATTTGCGT
